# Microspheres for 3D bioprinting: a review of fabrication methods and applications

**DOI:** 10.3389/fbioe.2025.1551199

**Published:** 2025-05-26

**Authors:** Dmitri Karaman, Kira Williams, Jolene Phelps, Fynn La Boucan, Gretchen Lewandowski, Kerrin O’Grady, Bosco Yu, Stephanie M. Willerth

**Affiliations:** ^1^ Department of Mechanical Engineering, University of Victoria, Victoria, BC, Canada; ^2^ Division of Medical Sciences, University of Victoria, Victoria, BC, Canada; ^3^ Axolotl Biosciences, Victoria, BC, Canada; ^4^ Biomedical Engineering Program, Department of Mechanical Engineering, University of Victoria, Victoria, BC, Canada; ^5^ Biology Program, Department of Science, University of Victoria, Victoria, BC, Canada; ^6^ Department of Biomedical and Chemical Engineering, Syracuse University, Syracuse, NY, United States; ^7^ BioInspired Syracuse, Institute for Material and Living Systems, Syracuse University, Syracuse, NY, United States; ^8^ School of Biomedical Engineering, Faculty of Applied Science and Faculty of Medicine, University of British Columbia, Vancouver, BC, Canada

**Keywords:** microspheres, bioprinting, biomaterials, emulsification, microfluidics, electrospray, drug encapsulation

## Abstract

Bioprinting incorporates printable biomaterials into 3D printing to create intricate tissues that maintain a defined 3D structure while supporting the survival and function of relevant cell types. A major challenge in 3D bioprinting is tuning material properties to ensure compatibility with different types of cells, while accurately mimicking the physiological microenvironment. Developing novel bioinks tailored to specific applications can help address this challenge by combining various materials and additives to tune the bioink formulation. Microspheres - small spherical particles - can incorporate drugs or growth factors to enable their controlled release, encapsulate cells to provide protection during printing, and provide structural reinforcement to tune mechanical properties and enable complex architectures. The particles range in size from 1 to 1000 μm and can be tuned to meet desired functions by optimizing their mode of production and the materials used for fabrication. This review presents an overview of microsphere production methods and considerations for optimizing the production process. It then summarizes how microspheres have been used to date in bioprinting applications. Finally, the existing challenges associated with the creation and use of microspheres are discussed along with avenues for future research.

## 1 Introduction

Biomaterials are biologically compatible materials that are designed to interact with biological systems and can help facilitate modern medical research by supporting or enhancing tissue growth and biological functions. Bioprinting combines 3D printing and the use of biomaterials to enable the production of complex tissues and tissue models that hold a defined 3D structure often containing applicable cell types and growth factors. When used in 3D printing, the combination of biomaterials and living cells are referred to as bioinks (Groll et al., 2018). Bioinks combine multiple materials and additives, that when 3D printed, mimic the complexity of the extracellular matrix and provide a structural framework to encourage cell growth and differentiation tailored to specific applications.

Developing biomaterials that are compatible with cells and the printing process while providing appropriate mechanical and functional properties that mimic tissues represent a major challenge in 3D bioprinting (Murphy and Atala, 2014). Material choices are further influenced by the ability to protect cells during the printing process. A promising additive that can help overcome these challenges are small spherical particles often referred to as microspheres, which can be incorporated into biomaterials to tune their biological and mechanical properties. Microspheres are uniform particles, with diameters ranging between 1–1,000 μm, and can be made from one or more materials (Li et al., 2018). In 3D bioprinting, microspheres have been used within biomaterials to encapsulate drugs or growth factors for controlled release (Poldervaart et al., 2014; Chen et al., 2020; De La Vega et al., 2021; Li et al., 2021; Bai et al., 2022; Wen et al., 2022; De Barros et al., 2023), improve the rheological properties of printed constructs (Tan et al., 2016; Xin et al., 2019; Sharma et al., 2021b; Wang W. et al., 2022; Mclean et al., 2024), as sacrificial particles to enable vascularization and/or migration of cells (Wang P. et al., 2022; Xie et al., 2022; Reynolds et al., 2023), and to encapsulate cells to protect them from shear forces and increase their viability during the printing process (Xu et al., 2019; Cohen et al., 2022; Chu et al., 2023; Ou et al., 2023; Yin et al., 2023). There is a need to review the technologies for microsphere production along with the bioprinting studies that take advantage of this technology.

Technologies for creating microspheres can be broadly classified into four main categories: mechanical agitation, membrane emulsification, microfluidics, and electrospray. Mechanical agitation describes one of the simplest and oldest technologies for generating microspheres and relies on large shear forces to mix the continuous and dispersed phases. Membrane emulsification extrudes the dispersed phase through a microporous filter and into the continuous phase. Microfluidic chips rely on fluid dynamics and a series of channels to direct the flow of different phases to create microspheres. Finally, electrospray relies on an electrical current passing through a syringe tip to generate microspheres. Current literature that applies microspheres in applications of bioprinting does not compare production methods but each application rather considers a single production method. Microspheres produced for use in printable biomaterials utilize the same techniques and materials as those used in other applications (e.g., as drug delivery vehicles), although with additional constraints for printability. Therefore, there is a need to identify existing technologies and methods of optimizing microsphere production that can be applied to improve current processes in the field.

This review provides a comprehensive comparison of the methods available for microsphere production and the applications of microspheres in 3D bioprinting. First, a summary of bioprinting techniques is provided. Next, methods of microsphere production are discussed, including their historical context. Further details are provided for each microsphere manufacturing technology, including their application in 3D bioprinting. Then, the literature utilizing microspheres for drug delivery, cell encapsulation, and as structural supports in 3D bioprinting is reviewed. Finally, gaps in the literature have been identified and recommendations are given for producing and using microspheres to improve 3D printed constructs as medical implants and as model systems.

## 2 3D bioprinting and bioinks

**FIGURE 1:**
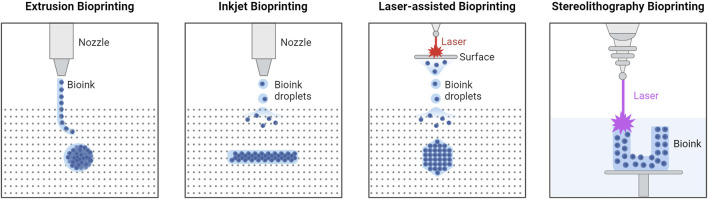
Types of 3D Bioprinting. Extrusion bioprinting uses mechanical forces to push a biomaterial out of the nozzle, relying on chemical or thermal crosslinking. Inkjet bioprinting uses either thermal deposit, electrostatic deposit, or piezoelectric actuators, and chemical or thermal crosslinking. Laser-assisted bioprinting uses a laser to illuminate a small section of the biomaterial layer, creating a high-pressure bubble that pushes the biomaterial layer to generate droplets that are then deposited onto the substrate. Stereolithography bioprinting uses UV light to crosslink the biomaterial layer-by-layer in the biomaterial reservoir. Created in BioRender. Willerth, S.M. (2024) https://BioRender.com/m18i644.

Bioprinting uses biological and bio-functional materials for additive manufacturing. Highly specialized printers are used to create 3D structures (i.e., tissue constructs) made from bioinks (McClements, 2023). A functional biomaterial for 3D printing should possess the following properties: printability (i.e., the ability to form and maintain a 3D structure when printed), high mechanical integrity to maintain its shape, insolubility in the culture medium so as not to degrade at physiological conditions, a controlled degradation rate, non-toxicity, non-immunogenicity, and if applicable, properties that promote cell adhesion (Ong et al., 2018). Biomaterial composition varies depending on the application, with most consisting of a viscous gel-like base (i.e., hydrogel) made from materials such as alginate, gelatin, gelatin-methacrylate (GelMA), poly(D,L-lactic-co-glycolic acid) (PLGA), and/or poly(ethylene glycol) (PEG). The concentration of the base material, addition of other materials (e.g., cells, microspheres, drugs, growth factors), and their interaction with each other is what produces the desired mechanical, biochemical, and physiological characteristics of the printed tissue constructs. Like traditional 3D printing, there are several techniques and types of printers that can be used depending on the material and desired final product. In 3D bioprinting, these techniques include extrusion, inkjet, laser-assisted, and stereolithography as can be seen in Figure 1; each with their own advantages and disadvantages as outlined below in Table 1.

**TABLE 1:** 3D bioprinting techniques: Advantages & disadvantages.

Printer type	Printing method	Crosslink method	Advantages	Disadvantages	References
Inkjet- Based	Thermal deposit, electrostatic deposit, piezoelectric actuators	Chemical or thermal	Low cost, high speed	Limited material viscosities	Bakhshinejad and D’souza (2015), Calignano et al. (2017), Gibson (2021), Vanaei et al. (2021)
Extrusion- Based	Mechanical Force by pistons, screws, or pneumatic pressure	Chemical or thermal	Large-scale structures created quickly	Low resolution, cell viability can be compromised due to shear stress caused by the pressure applied through the nozzle	Bakhshinejad and D’souza (2015), Calignano et al. (2017), Gibson (2021), Vanaei et al. (2021)
Laser- Assisted	Laser illuminates a small section of the biomaterial layer, creating a high-pressure bubble. The bubble then pushes the biomaterial layer while generating droplets deposited onto the substrate	Laser cured	Droplets can be deposited with high resolution and viscosity	Potential for cell damage due to the laser intensity, high cost, limited materials	Bakhshinejad and D’souza (2015), Calignano et al. (2017), Gibson (2021), Vanaei et al. (2021)
Stereo-lithography	Biomaterial in reservoir, crosslinked layer-by-layer to create construct	UV light	High resolution, can generate complex geometries	Biomaterials must be photosensitive, cell damage due to light, high biomaterial waste	Bakhshinejad and D’souza (2015), Calignano et al. (2017), Gibson (2021), Vanaei et al. (2021)

Regarding applications that incorporate microspheres into bioprinting, most of the literature has relied on extrusion bioprinting, although microspheres can be incorporated into many bioprinting technologies (Lee et al., 2011; De La Vega et al., 2018; Yao et al., 2019). Printability of the biomaterial is an important consideration for the addition of microspheres and depends on both the printing technology and microsphere composition. Flow characteristics within technologies that dispense biomaterial via a nozzle, including extrusion, inkjet, and microfluidic bioprinting, are impacted by viscosity and particle size (Chen, 2025). The inclusion of microspheres will impact the rheological properties of biomaterials, affecting the flow characteristics (Levato et al., 2014). Photopolymerization techniques rely on light transmission for crosslinking which will be affected when including additional particles (Gentry and Halloran, 2015).

## 3 Methods of microsphere production

Primary factors that must be considered for microsphere production in 3D bioprinting include the application (i.e., whether for drug delivery, cell encapsulation, or for structural considerations), bioprinting technology (e.g., extrusion, stereolithography, inkjet, or laser assisted), microsphere composition, desired size and/or variability in size, and the amount to be produced. Microsphere generation methods vary in their level of control, throughput, and chemical compatibility. In many cases, more than one method will be applicable to the identified needs. Microspheres are commonly synthesized using two immiscible phases, energy to create an interface between the two phases, and surfactants or cross-linkers to stabilize this interface to prevent the drops from coalescing immediately upon contact and to form the microspheres (Rayner and Dejmek, 2015). The two phases are referred to as the dispersed and continuous phases, where the dispersed phase makes up the polymeric solution that forms the microspheres and the continuous phase is the external solution which suspends the microspheres. The four main categories for producing microspheres are outlined in Table 2, along with their advantages and disadvantages. The historical context of microsphere manufacturing methods, and details of the outlined production methods are described in the following sections, including parameters for tuning generated microspheres, the types of specialized equipment needed, and associated costs and considerations.

**TABLE 2:** Advantages and disadvantages associated with the most commonly used methods for generating microspheres.

Method	Principle	Advantages	Disadvantages	References
Mechanical agitation	Stirring of two insoluble phases forms an emulsion, and subsequent crosslinking or solvent evaporation forms microspheres	Low complexity, can be done with classical lab supplies without the need for complex equipment, easy to scale up	Addition of surfactants necessitates a complex cleaning process, poor encapsulation efficiency of hydrophilic molecules, Less control over the size and shape of microspheres	Li et al. (2008), Iqbal et al. (2015)
Membrane emulsification	Pressure through microporous filters generates microspheres	Creates consistently sized microspheres, high throughput, low energy input	Low dispersed phase flux through the membrane, clogging of filters inhibits production, size dependent on filter parameters	Hancocks et al., 2013; Ma (2023)
Microfluidics	Complex system of microchannels where fluid flow creates droplets	Creates uniformly shaped and sized microspheres, highly tunable parameters and very reproducible	Expensive and complicated to produce, time consuming to operate, many factors for operation	Zhang et al. (2019)
Electrospray	Pre-mixed solution is sprayed through a nozzle and high voltage at outlet forms liquid droplets	Creates uniformly sized microspheres with tight control of size distribution, does not require addition of surfactants	Low yield, complexity, not ideal for high viscosity solutions due to high electrostatic forces that need to be overcome	Tapia-Hernández et al. (2015), Chi et al. (2023)

### 3.1 Historical context of microsphere manufacturing technology

The development of technologies for microsphere generation builds on decades of progress in related fields. Equivalent production methods are currently used in a number of industries including cosmetics, food additives, and pharmaceuticals (Rayner and Dejmek, 2015). Mechanical agitation is well suited to large-scale manufacturing due to its high yield, while methods such as microfluidics offer a finer degree of control (Li et al., 2018). High precision emulsions, including those created for pharmaceuticals, can fulfil the requirements for microspheres used in bioprinting, namely, biocompatibility, narrow size distribution, and controlled degradation.

The basic principle behind microsphere production relies on creating an emulsion with consistent and small particle sizes, a process that has long been researched in food production (Rayner and Dejmek, 2015). An early example of an oil-in-water (O/W) emulsion is mayonnaise, which exhibits higher structural stability and viscosity relative to the precursor materials (Liu et al., 2018). The high-energy agitation of the two components disperses the oil phase to create smaller particles in a stable emulsion, using lecithin as an emulsifying agent. This process of mechanical agitation is the same process regularly used for microsphere production (Rayner and Dejmek, 2015). In 1947, uniform soap bubble microspheres were used to model crystalline structures (Bragg and J.F., 1947), where later experiments used microbubbles to create porous metal foams (Ashby et al., 2000). Bragg and J.F. experimented with fluid dynamics and surface tension, reducing microbubble size with a rotary flow in the continuous phase, a technique currently utilized in membrane emulsification. Microfluidic channels and membrane emulsification techniques are both utilized for food-based emulsifications and were later repurposed to create microspheres for use in biomedical applications (Rayner and Dejmek, 2015). As emulsifications saw increased demand and need for quality, many methods of production have been developed. Most microsphere production methods took advantage of the decades of research into food emulsifications that preceded them, and much of this knowledge is still relevant to current microsphere production (Katoh et al., 1996). Some novel production methods, such as electrospray and 3D printing have fewer historical sources to draw knowledge from.

### 3.2 Mechanical agitation

Mechanical agitation methods mechanically mix two immiscible solvents to form an emulsion, and then crosslinking is initiated by either the addition of specific substances, evaporation of a solvent, or a change in temperature or pH, to prepare stable microspheres. Mechanical mixing in a research lab setting is commonly done using a magnetic mixer, where a magnetic stir bar is placed inside a beaker, and a separate magnet underneath the container is attached to a motor. Magnetic mixers are limited to small volumes (i.e., <4 L) and low viscosity suspensions. For larger volumes or more viscous liquids, an overhead stirrer can be used, which employs a bladed shaft. An overhead stirrer is limited to speeds of about 3,000 rpm but can work with a high range of viscosities. For the generation of smaller micro- and nano-sized particles, homogenizers can be used to generate higher shear forces. Different models of homogenizers use different physical technologies, including standard blender-type instruments, bead mills, ultrasonication, high pressure, or other physical forces.

**FIGURE 2:**
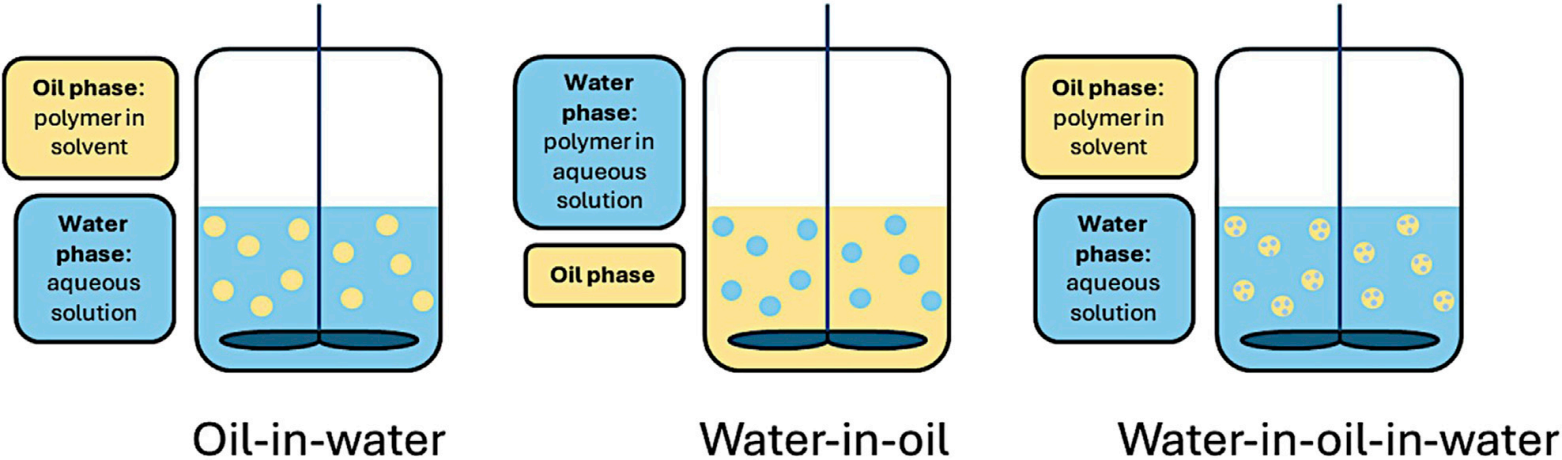
Mechanical agitation using oil-in-water (O/W), water-in-oil (W/O), and double emulsion water-in-oil-in-water (W/O/W) emulsion methods.

Each of the two solutions can be referred to as an oil phase or a water phase depending on the polymer(s) selected and the solvent used. If the polymer is dissolved in an aqueous solution, the dispersed phase is referred to as a water-phase, and if the polymer is only soluble in an organic solvent, the dispersed phase is referred to as an oil phase. Emulsions can generally fit into 3 categories: oil-in-water (O/W), water-in-oil (W/O), or double emulsion water-in-oil-in-water (W/O/W), as described below, and seen in Figure 2. More recently, water-in-water systems have been developed, referred to as aqueous two-phase systems. The choice of emulsion method depends on the solubility of the polymer, application, and if used for drug delivery, the characteristics of the drug (e.g., hydrophobic vs. hydrophilic). Though other methods exist, including oil-in-water-in-oil (O/W/O), and solid-in-oil-in-water (S/O/W), they are less common for microsphere production in health research and will not be detailed in this review.

#### 3.2.1 Oil-in-water single emulsion (solvent evaporation)

Single emulsion solvent evaporation, often referred to as O/W emulsion, is a commonly used technique for drug encapsulation of insoluble or poorly water-soluble biomolecules. A polymer solution (oil) is added into an aqueous solution (water) containing a surfactant or stabilizer. The most commonly used surfactant is poly (vinyl alcohol) (PVA). The polymer (e.g., poly(lactic-co-glycolic acid) (PLGA)) is first dissolved in an organic solvent, most commonly dichloromethane (DCM), also containing the drug for encapsulation. The emulsion is formed mechanically through stirring or by using a homogenizer. The solvent is then removed by evaporation, typically overnight. While DCM is the most commonly used solvent due to its high volatility, low boiling point, and high immiscibility with water, it is a confirmed carcinogenic according to the Environmental Protection Agency and therefore should be used with caution depending on the application of the microspheres (Schlosser et al., 2015). Additional constituents, referred to as co-solvents, can be added to the dispersed phase to help dissolve drugs that are not soluble in the chosen solvent, or to generate pores inside the microspheres (e.g., hexane).

Encapsulation efficiency, size, morphology, and drug loading can be tailored by altering the polymer to solvent ratio (i.e., wt% of polymer dissolved in the solvent), stir or homogenizing speed, drug concentration, stabilizer concentration, and other formulation parameters. For example, increasing the polymer concentration, and thereby the viscosity of the oil phase, results in the formation of larger particles, while increasing the speed of agitation reduces the particle size (Zhang and Gao, 2007). Changes to viscosity can also alter the encapsulation efficiency (Li et al., 2008). Increased polymer concentration has been found to increase encapsulation efficiency and slow drug release (Yang et al., 2000). The stabilizer concentration is another important factor, with increased concentrations leading to reduced particle size and lower drug encapsulation efficiencies (Floyd et al., 2015). This is attributed to decreased surface tension between the oil and aqueous phases, and increased aqueous viscosity, reducing the size of the particles, and leading to loss of drugs into the aqueous phase.

O/W emulsions suffer from lower encapsulation efficiencies for hydrophilic compounds, as these preferentially diffuse out from the oil dispersed phase into the aqueous continuous phase. In the case of highly hydrophilic compounds, drug encapsulation is not possible in O/W emulsions (Iqbal et al., 2015). This can be overcome by oversaturating the aqueous phase with the drug (Zhang and Gao, 2007), by using a W/O emulsion if the polymer can be dissolved into the aqueous phase, or through the use of more complex double emulsions.

#### 3.2.2 Water-in-oil single emulsion

W/O emulsions are created when the polymer is dissolved in the aqueous phase and is added into an oil bath. For example, gelatin microspheres can be synthesized by dissolving gelatin in water and adding it into a continuous phase of paraffin oil with or without the addition of a stabilizer (e.g., Span 80) (Poldervaart et al., 2014). The microspheres must then be crosslinked, which can be done through the addition of a photo initiator followed by UV exposure, or the addition of a crosslinking agent after the emulsion is formed (e.g., glutaraldehyde). The drug is loaded into the aqueous phase for drug loading applications. W/O methods can also be used for encapsulation of cells, with cells loaded into the aqueous dispersed phase in either a phosphate buffered saline (PBS) or cell medium (e.g., Dulbecco’s Modified Eagle Medium, DMEM) based buffer, however this is more commonly done through the use of microfluidic chips, or other methods that do not expose the cells to high levels of shear (see Section 3.3).

#### 3.2.3 Water-in-oil-in-water double emulsion

Double emulsion solvent evaporation, often referred to as W/O/W emulsion, is a method that can be used to overcome the reduced encapsulation efficiencies for hydrophilic compounds in O/W emulsions. For example, the encapsulation efficiency of acetaminophen was 3 times higher using a W/O/W emulsion as compared to an O/W emulsion, while also showing more sustainable release characteristics (Lai and Tsiang, 2005). Using a W/O/W method, the drug is dissolved into an aqueous solution and emulsified into an organic solvent to form a W/O emulsion. The W/O emulsion is then added to a second aqueous solution containing the stabilizer and emulsified again as is done for an O/W emulsion. The additional step enables the organic layer to act as a barrier that prevents the drug from diffusing into the external aqueous phase. As for O/W emulsions, the solvent is evaporated overnight prior to microsphere recovery. Double emulsions typically produce more polydisperse particles compared to other techniques, but offer the advantages of being able to entrap both hydrophobic and hydrophilic molecules with high efficiencies, protecting them against light, enzymatic degradation, and oxidation, and enabling slow and sustained release of the molecules (Iqbal et al., 2015).

Similar to single emulsion techniques, formulation parameters can be tuned to alter encapsulation efficiency, drug loading, particle size, and morphology. Encapsulation efficiencies can generally be improved with higher polymer concentrations, though this is dependent on the encapsulated drug (Shah et al., 2014). Higher polymer concentrations stabilize the O/W interface and result in particles with a more dense matrix and decreased surface porosity, leading to reduced water penetration and therefore reducing drug leaching as the microspheres are formed (Shah et al., 2014). The stability and release properties of double emulsions can also be improved by changing the type and concentration of stabilizers. For example, the conformational stability and encapsulation efficiency of a monoclonal antibody, 3D8 scFv, was significantly improved by the selection of an appropriate stabilizer, mannitol (Son et al., 2009), highlighting the need for process optimization in microsphere production.

Overall, mechanical agitation methods can produce microspheres capable of encapsulating drugs and cells, with tunable properties such as size, stiffness, and porosity determined by the agitation speed, and the composition of the dispersed and continuous phases. The choice of emulsion method is determined by the desired use of the microspheres. The above-mentioned emulsion types can be applied in more complex production techniques that can produce more consistently sized and shaped microspheres, described in the following sections.

#### 3.2.4 Aqueous two-phase systems (water-in-water emulsion)

Aqueous two-phase systems (ATPS) rely on water-in-water emulsions and can be used to generate microspheres. The use of a water-based system limits the inactivation of drugs during the manufacturing process and therefore may be of benefit to drug encapsulation applications, if the drug is soluble in an aqueous solution. Typical manufacturing often relies on the use of organic solvents, which are not only toxic and harmful to the environment, but may reduce the efficiency of drug loading and sustained drug delivery processes (Mizukami et al., 2022). This form of microsphere production shows benefits as an effective method of drug-loading for microspheres but has seen minimal use in the space of 3D bioprinting. The relatively large size distributions of ATPS-formed microspheres (Li et al., 2020; Mizukami et al., 2022) may reduce efficiency in printability and affect printability but is worth considering for future research. ATPS differs from other emulsions as the microspheres or microgels can be created directly within a hydrogel biomaterial rather than added afterwards (Wang Q. et al., 2023).

### 3.3 Membrane emulsification

Membrane emulsification is a commonly used method of microsphere production at both consumer and industrial scale. This broad category uses pressure through microporous filters to generate microspheres. Unlike mechanical agitation, membrane emulsification is primarily reliant on interfacial tension and applies lower forces to the microspheres (Rayner and Dejmek, 2015). The pressure is applied to the dispersed phase to force it through the filter and as it exits the other side, it interacts with the continuous phase and creates droplets (Dragosavac et al., 2012). There is significant variety in filter shape, size, and composition, with each having different characteristics during microsphere production (Joscelyne and Trägårdh, 2000; Flanc et al., 2023). The shape and size will affect the maximum throughput and possibility of clogging, and the composition primarily affects pore shape. Pores with the same size but different compositions can produce substantially different microsphere sizes (Hancocks et al., 2013). Membrane emulsification can provide repeatable microsphere sizing and have high throughput relative to other methods. Due to this, it is commonly found in both scientific and industrial settings (Hancocks et al., 2013; Ma, 2023).

**FIGURE 3:**
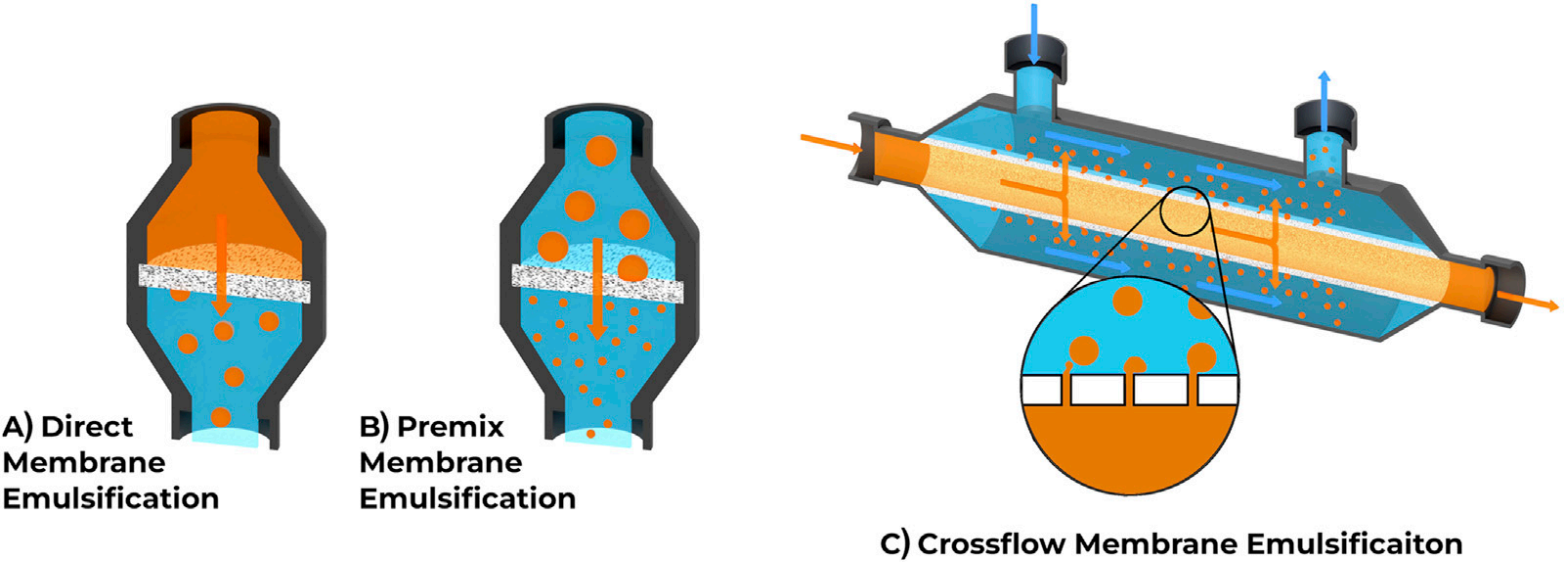
Comparison of common membrane emulsification techniques. **(A)** Direct membrane emulsification uses a single pass through the membrane to create microspheres. **(B)** Premix membrane emulsification involves a solution with larger microspheres being passed through the membrane to decrease microsphere size and increase consistency, often repeated several times. **(C)** Crossflow emulsification uses a different membrane orientation and shape, allowing phases to flow parallel to the membrane.

Filters typically come in one of two forms: disc filters and cylindrical filters. The choice of filter depends on the filtering method, which is described in more detail in the following sections. Direct and Premix membrane emulsification often use a flat disc or rectangular filter in which the dispersed phase flows through the filter, as seen in Figures 3A,B. Direct membrane emulsification uses a single pass through the filter to create microspheres and tends to have a wide particle size distribution (Rayner and Dejmek, 2015). Premix emulsification starts with an initial emulsion with a wide particle distribution which becomes narrower and more consistent in subsequent passes. Cross-flow emulsification uses cylindrical or pipe shaped filters, where material flows parallel to the filter and often requires less pressure (Hancocks et al., 2013), as seen in Figure 3C. The mechanisms for generating microspheres differ between the two methods. Cross-flow filtration uses fluid flow in the continuous phase for the detachment of the microspheres while dead-end filtration typically uses a stationary continuous phase and relies strictly on the interfacial tension in each microsphere. Dead-end filtration systems can be more affordable and require less specialized equipment. For higher throughput requirements, cross-flow filtration methods can be beneficial due to their larger surface area, meaning more simultaneous sphere production and less risk of filter clogging.

The main types of filters utilized for membrane emulsification are Shirasu Porous Glass (SPG), sintered metal, microsieves/laser drilled metal, and glass fiber filters. The pore sizes and geometry can vary greatly depending on the filter material (Hancocks et al., 2013). Two materials with similar pore size but different geometry will often create different sized microspheres. SPG filters are the most used in literature due to their consistent pore structure and size. These filters are made with volcanic ash and can be formed into different shapes (*i.e.*, flat or as cylindrical membranes), while having strong chemical, temperature, and pressure resistance. Microsieves and other laser etched or drilled filters have the most accurate control over membrane pore size and shape, with each pore being directly created. Unlike other filter types, these have no complex internal structure, and the pores go straight through the material. The opposite side of the spectrum is glass fiber filters which are often used to create larger microspheres due to the pore closeness, where multiple microspheres coalesce and form larger structures (Hancocks et al., 2013). Membrane emulsification can have additional steps taken either during the creation of the microspheres or afterwards. For example, Mugabi et al. highlights the use of swirling fluid flow within the cylindrical filter to modify the forces applied on the microspheres (Mugabi et al., 2019). Similar steps have been taken with flat filters to assist with microsphere detachment (Dragosavac et al., 2012; Ohta et al., 2018).

#### 3.3.1 Direct membrane emulsification

Direct membrane emulsification is the most basic method of membrane emulsification, utilizing a flat filter and a single pass for creating microspheres. The dispersed phase flows from one side of the filter to the other side, which contains the continuous phase. This method has few components for set-up and can produce large volumes of microspheres due to its simple operation. Although small microspheres can be produced, they tend to have a large variance in size unless other steps are taken.

Direct emulsification has been utilized for tissue engineering with human hemoglobin and albumin based microspheres (Ohta et al., 2018). Some mixed methods use direct emulsification together with mechanical agitation to increase flow forces at the surface of the filter. This method is sometimes referred to as rotary flow emulsification, as it adds a rotational force to the continuous phase (Rayner and Dejmek, 2015). Camelo-Silva *et al.*, use a stainless-steel filter and stirring device to encapsulate a probiotic culture, along with Sodium Alginate, Whey Protein, Rice Protein, and Pea Protein (Camelo-Silva et al., 2023). Spherical silica particles were created with a similar set-up, and resulted in 30–70 µm particle sizes (Dragosavac et al., 2012). Yeast have been encapsulated with this technique, showing the possibility of cell-encapsulation, although current research on this topic is limited (Morelli et al., 2017). Direct membrane emulsification can be utilized with a wide range of applications and materials, but the variation in particle size is one limiting factor for its use. Swirl flow, or rotary flow helps achieve narrower size distributions and creates a system closer to crossflow techniques.

#### 3.3.2 Premix membrane emulsification

Premix membrane emulsification uses the same basic form of direct membrane emulsification, but adds multiple passes of it. It involves pressing the dispersed and continuous phases through the flat disc membrane to produce smaller, and more consistently sized particles with each pass. Premix membrane emulsification begins with a coarse emulsion, either created externally with a vortex or homogenizer, or done in the first few passes of membrane emulsification. The number of passes through the device varies based on the set-up. Due to the pore size of the filters, there is a minimal size that the particles will trend towards. Further passes will achieve microspheres that are more similar in size and closer to the minimum possible size. The initial passes will have the greatest effect, with diminishing returns each pass after. Each filter material has a different ratio of pore size to minimum particle diameter, though most filter types can produce much smaller microspheres than the actual pore size.

Premix emulsification requires minimal equipment and set-up and can be done with a filter, filter holder, and syringe, making it cost effective relative to other techniques. The cost of the filters and filter holders can vary based on the filter material and required chemical resistance. It is important to ensure chemical compatibility of each of the components as commonly used solvents such as DCM can be incompatible. Filters holders can be made from a wide range of plastics and metals and will typically use an O-ring for pressure sealing. These, along with the syringes themselves, will have a subset of materials that can be used without degrading.

Premix membrane emulsification is used extensively for drug delivery and works with a wide range of reagents and drugs. Often, a biodegradable polymer such as polylactic acid, polycaprolactone, or poly (lactic-*co*-glycolic acid) will serve as a base material for drug encapsulation and delivery (Ma, 2023). Ma (2023) highlights an extensive list of drug-loading materials used with premix emulsification including ropivacaine and chitosan. This book provides a comprehensive review of current membrane emulsification literature.

#### 3.3.3 Cross-flow membrane emulsification

Crossflow membrane emulsification utilizes fluid shear force to separate microspheres from the membrane surface. The continuous phase flows perpendicular to the membrane and this method typically utilizes cylindrical membranes. Cross-flow membrane emulsification is a useful method for scaling up microsphere production. It reduces the pressure exerted on microspheres as they are generated, increasing consistency and minimizing shear stress. The size, shape, and direction of flow for cylindrical filters reduces the risk of clogging and drastically increases the number of pores available for producing microspheres. Instead of a flat disc with an inlet and outlet, a cylinder can be used with the inner wall making up the inlet, and the outer surface acting as the outlet. The main difference in cross-flow membrane emulsification is the flow of the continuous phase. The addition of a fluid shear force along the outer surface of the membrane helps detach each microsphere with less force being applied to the dispersed phase. Lower forces can help create more consistent microspheres while being less likely to damage the microspheres or cells loaded within them.

Setting up a cross-flow emulsification system requires more specialized equipment than a premix emulsification system. Cross-flow emulsification systems require separate pressure/flow controllers for continuous and dispersed phases, along with larger volumes of each, limiting the use of certain flow control systems. Due to the cylindrical shape of the membrane, a different type of filter holder is required to accommodate the separate inner and outer flows. The cost of equipment can be prohibitively expensive for smaller scale operations, making it more suitable for labs and companies regularly producing microspheres at a larger scale. The materials that can be used for cross-flow emulsification are the same as other membrane-based methods. A wide range of materials have been used including polyethylene glycol, poly(d,l-lactide-*co*-glycolide), and haloperidol (Meyer et al., 2010; Henise et al., 2021). Cross-flow emulsification has developed microspheres with other common biomaterials such as polycaprolactone and polypropylene(Rayner and Dejmek, 2015).

### 3.4 Microfluidics

Microfluidic systems rely on complex systems of channels and junctions to control fluid dynamics. Three main structures have been utilized in microfluidic designs to generate microspheres: co-flow, cross-flow, and flow-focusing (Zhang et al., 2019). The creation of microspheres is highly dependent on the channels and channel junctions as opposed to the flow rate and fluid properties (Abate et al., 2009). This review will primarily discuss passive microfluidic generation, where the fluid flow creates droplets. Other methods combine the passive system with ultrasonic, electrical, thermal, or other methods to increase efficiency and control (Zhang et al., 2019).

Microfluidic chips enable a higher degree of control not observed in alternative microsphere production methods. Each microsphere can be produced individually, with full control over each parameter during the production (Chi et al., 2023). Many chip designs are transparent, allowing sphere formation to be directly monitored (Chen et al., 2020). The produced microspheres have a highly uniform shape and size that can be tuned with many parameters (e.g., flow rate, channel size, junction design, material composition), and some microfluidic chip designs can even be adjusted during operation. The wide range of parameters gives microfluidic chips the most control, while also having the highest complexity to operate. Creating microfluidic chips can be difficult due to the complex fluid characteristics within them, and manufacturing is often time-consuming and expensive. Recent advances have allowed the use of 3D printing in chip generation, which makes the chips easier to produce and reduces manufacturing costs. Although additive manufacturing has made microfluidics more accessible, it can still be difficult to produce accurate channels at high resolution (i.e., <50 µm), which is necessary to produce smaller microspheres (<10 µm).

Microfluidic systems are highly flexible in their application due to the number of adjustable parameters through channel design, construction material and method, and material flow rates. Because so many factors can be modified, they can be used for any application and are regularly used for drug delivery and cell encapsulation. Microfluidic chips are used with a number of natural macromolecules and synthetic polymers including alginate, gelatin, chitosan, polyglycerol, poly(ethylene glycol), and many more (Li et al., 2018; Zhang et al., 2020). Additionally, many studies have shown successful use with cell encapsulation, encapsulating engineered cardiac tissue, glioblastomas, and colorectal cancer cell lines (Zhang et al., 2022; Finklea et al., 2024). The main limiting factors for microfluidic based production are lower throughput and a high number of variables to adjust compared to alternate methods.

#### 3.4.1 Microfluidic chip design

**FIGURE 4:**
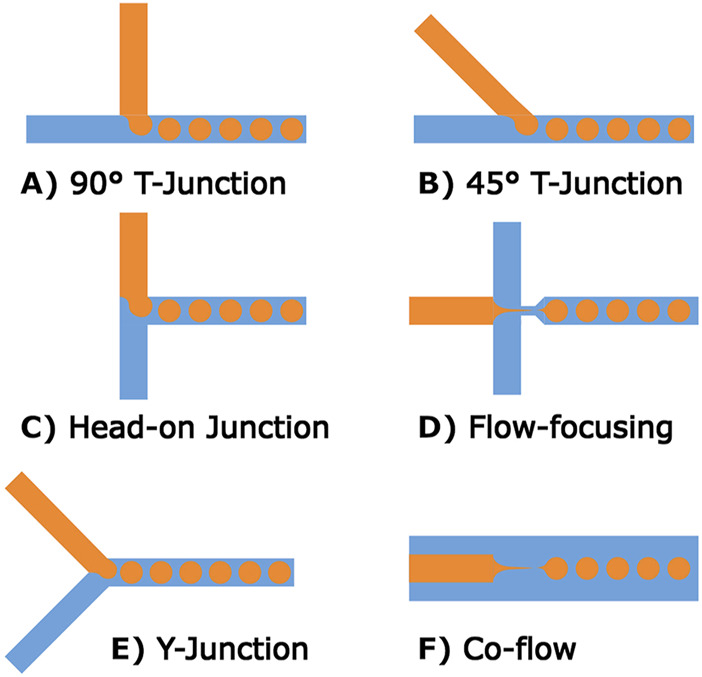
Common microfluidic channel designs for microsphere production. **(A–C)** Show different forms of T-junctions, these channel designs produce stable microspheres at low-medium rates. **(D)** A flow-focusing method where the central channel is narrowed at the outlet. **(E)** Shows a variation of T-junction geometry referred to as a Y-junction. **(F)** A different form of flow-focusing geometry relying on a co-axial geometry.

In microfluidic chips, the channel sizes and shapes, along with junction types, have an effect on the forces utilized in production, although few direct comparisons exist between junction types. Broadly speaking, larger channels result in larger microspheres and smaller channels enable the production of smaller microspheres. However, smaller channels become more difficult to produce and can prohibit certain manufacturing methods. For example, creating channels smaller than 50 µm in diameter is difficult for most methods of 3D printing. The main junctions used in the literature are co-flow, crossflow (T-junctions, Y-junctions) and flow-focusing as shown in Figure 4, with a wide range of additional junctions and variations available (Zhang et al., 2019). Different types of junctions have their own set of operating parameters, such that optimal flow rates for one junction will not necessarily apply to other junction types. Some advanced junctions are mechanically, chemically, or electrically controlled and their channel size can be adjusted based on user input; however, these are less frequently used (Zhang et al., 2019). It is also possible to create microspheres using different phase parameters (O/W, W/O, W/W, W/O/W, O/W/O) with microfluidic chips, making them a versatile option for highly controlled microsphere production.

#### 3.4.2 Microfluidic chip manufacturing methods

Depending on the type of microfluidic chip and the size of channels, there can be many methods of production with their own advantages and disadvantages. Manufacturing in-house has the shortest lead-times and allows the greatest flexibility for generating new designs. All production methods can be outsourced or created in-house, although some are more readily accessible than others. Each manufacturing method can be used for microsphere production, but the exact composition and size of microspheres will determine which manufacturing method is best suited for production. Factors to consider when choosing a manufacturing method are the required microfluidic chip material, minimum channel size, production number, and cost.

Traditionally, microfluidic chips were made using photolithography and either etched glass or silicone wafers (Ren et al., 2013). Photolithography uses a 2D pattern called a photomask, and a surface coating called a photoresist. After coating the surface with the photoresist and adding the mask on top, a powerful light cures the desired areas and the rest of the photoresist can be washed off. The areas not covered are etched to produce the channels. Photolithography is still used as it can produce channels with a high degree of accuracy (Ren et al., 2013). Soft lithography uses photolithography to create a negative of the microfluidic channel and uses it as a mold instead of the final product. With this negative, a polydimethylsiloxane (PDMS) microfluidic chip can be produced by curing it and removing it from the mold (Grösche et al., 2019). PDMS chips can be affordably produced after the initial mold is created, reducing the total cost for the system. However, the hydrophobic nature of PDMS can cause some challenges, and may require additional surface treatment to function correctly. The flexibility of PDMS can also cause additional challenges when higher pressures are applied, making it unsuitable for certain designs.

Hot embossing is a comparable technique to soft photolithography as it uses a negative of the microfluidic chip to create the final chip. In this case, the negative is used as a stamp and heated up before being pressed into a thermoset polymer such as polymethyl methacrylate (PMMA) (Wang et al., 2011). After cooling the materials, the stamp is separated from the final piece and ready for use. This technique is more expensive than soft lithography as it needs a specialized press system that can maintain the required temperature. There is a wide range of thermoset materials that can be utilized for this purpose such as the previously mentioned PMMA, cyclic olefin polymer (COP), and polycarbonate (PC).

A more specialized and expensive method of production is injection molding. While it can mass-produce microfluidic chips at a low cost, the design and production of molds typically requires high accuracy metal fabrication. The design of molds has many unique challenges such as maintaining consistent material flow during casting, and ensuring the mold is possible to produce with the given manufacturing method (Etxeberria et al., 2024). A new mold must be made for any channel changes, making it most applicable for large-scale manufacturing finalized designs.

The newest method of manufacturing, and often the most accessible, is 3D printing of microfluidic devices. Due to the large expansion of the consumer 3D printing market, low-cost 3D printers have become significantly more affordable and accessible. Both fused deposition modeling (FDM) and masked stereolithography (MSLA) printers can now be purchased from large retailers such as Amazon and Walmart for ∼$150 USD, making them more accessible than ever before. Some functional FDM microfluidics have been produced but they have larger channel sizes than generally desired and a rougher surface finish. Other printing methods, especially those utilizing photopolymers, can produce higher resolutions and smooth surface finishes (Grösche et al., 2019). MSLA, stereolithography, directed light projection, and polyjet all utilize photopolymers for their manufacturing, with all but polyjet available as consumer products. Binder jetting, material jet fusion, and selective laser sintering have high resolutions but are powder-based technology, leaving a rougher surface finish. The microfluidic chip can either be directly printed or a negative can be printed for other production methods. For lab settings where many variations of channel designs are required, this is likely the best solution. The main downsides are the lack of material options and the minimum channel size depending on the type of printer being utilized (Zhang et al., 2019).

### 3.5 Electrospray

Electrospray is an effective and precise technique for microsphere production. To create microspheres using the electrospray method, the dispersed phase is extruded from a syringe into droplets and exposed to an electric field, thereby forcing the droplet to split into microspheres via electrostatic forces (Qayyum et al., 2017; Chi et al., 2023). The electric field is created by connecting positive and negative electrodes to the syringe and collector respectively (Chi et al., 2023). Once collected, the microspheres are formed by evaporation and/or by exposing them to different conditions or compounds to induce crosslinking during the spray process (e.g., photo-initiated, thermo-crosslinked) or in the collector (e.g., CaCl_2_). Electrospray enables the production of uniform microspheres with homogenous structure with higher efficiency, compared to emulsion and microfluidic techniques (Qayyum et al., 2017). In addition, the electrospray technique can avoid the use of organic solvents like DCM, which are commonly used in solvent evaporation to confer microsphere stability, and may degrade encapsulated drugs and prohibit their use for cell encapsulation (Arya et al., 2009; Morais et al., 2020). Electrospray without organic solvents as a W/O method also avoids the solvent removal step following emulsion techniques, increasing the loading ability of microspheres compared to those with solvents due to the extensive number of washes required for solvent removal (Morais et al., 2020).

The physical characteristics of the microspheres can be adjusted by modifying the voltage of the electric field, the flow rate from the syringe, the viscosity of the polymeric solution, type of solvent, temperature and humidity, nozzle type, the distance to the collector, and the type of collector used (Morais et al., 2020). Higher concentrations of polymers (i.e., higher viscosity solutions), tend to form fibers as opposed to particles due to the higher level of electrostatic forces required to break the surface tension of the solution (Morais et al., 2020). Similarly, increasing the voltage of the electric field can lead to fiber formation and irregular particle morphologies (Correia et al., 2014). The type of solvent used alters microsphere production due to differences in electrical conductivity, surface tension, viscosity, dielectric constant, and evaporation rate or volatility. Microspheres of smaller size can be generated from solutions with higher electrical conductivity for example. Flow rate can influence the porosity and size distribution of microspheres, where high flow rates lead to larger particles and result in impaired evaporation and inconsistent morphologies, and low flow rates reduce particle size (Faramarzi et al., 2016).

Microspheres created using electrospray can be loaded with pharmaceutical agents and other biologically relevant compounds. Encapsulation is achieved by adding the compound to the dispersed phase or in some cases, the continuous phase prior to electrospray (Qayyum et al., 2017). For example, Mirek et al. (2023) loaded polycaprolactone (PCL) and polyethersulfone (PES) microspheres with antibiotics rhodamine or ampicillin in the continuous phase. The polymer solution was pumped through a metal nozzle with high pulsed voltage, and collected in a precipitation bath containing a polymer non-solvent and the antibiotics to undergo wet phase inversion. More commonly, compounds are added into the dispersed phase and undergo solvent evaporation during the spray process, as done for rifampicin in PLGA (Hong et al., 2008), drugs in PLA (Valo et al., 2009), and bovine serum albumin in PLA (Xu and Hanna, 2006). In addition, microspheres created via the electrospray method are compatible with cells and can be added to bioinks or biomaterials to improve the mechanical properties of 3D-printed scaffolds. For example, Xin et al. (2019) created PEG microspheres using electrospray which were photo crosslinked using UV light in a bath of mineral oil with Span 80. The electrospray microspheres were made with different stiffnesses to evaluate growth of mesenchymal stem cells (MSCs) and showed good biocompatibility, and with the addition of RGD, enabled cell attachment. They were then added to 3D-printed structures to increase the structures stability. Cells have also been successfully incorporated into the dispersed phase during electrospray. Wu et al. (2022) developed an organ-on-a-chip liver model using cell-laden microspheres created using the electrospray method. Here, liver cancer cells and endothelial cells were added to 2% sodium alginate and incorporated into microspheres via electrospray, crosslinked in a calcium chloride (CaCl_2_) bath.

While electrospray can create uniformly sized microspheres and can support their production without the use of harsh solvents, it is limited by low microsphere yield, complexity, and the use of low viscosity solutions as high viscosity solutions require too high of electrostatic forces to generate the droplets (Tapia-Hernández et al., 2015; Chi et al., 2023). Overall, the choice of microsphere generation technique is dependent on the scale of production, type of polymer, characteristics of the drug to be loaded if applicable, and the application for which it is to be used within bioprinting.

While most methods of creating microspheres can be applicable to each application, tailoring the production methods to the application will help improve the desired final outcomes, whether that be high encapsulation efficiency of drugs, increased cell viability and proliferation, high yields, low cost, or consistent sizing.

## 4 Applications of microspheres in 3D bioprinting

Early studies described the use of cell-laden microspheres in bioprinting, which were found to reduce the initial cell density required for bioprinting and improve the compressive strength (Levato et al., 2014; Tan et al., 2016) Further studies have described the use of microspheres for local delivery of drugs such as small molecules (Li et al., 2021) and growth factors (Wen et al., 2022; De Barros et al., 2023), for encapsulation of up to four cell types (Wu et al., 2022), to provide structural support (Wang W. et al., 2022), and as sacrificial particles to create porous networks (Wang P. et al., 2022; Xie et al., 2022; Reynolds et al., 2023). Bioprinted tissues containing microspheres have been used in applications for tissue regeneration, drug screening, and modeling tissue and organ systems including neural, liver, lung, breast, and skin tissues. Table 3 summarizes applications in which microspheres have been used in bioprinting applications, including details on how they were produced. The following sections detail the use of microspheres in 3D bioprinting for localized and sustained release of drugs and/or growth factors (referred to as drug delivery), encapsulation or attachment of cells (cell laden), and as structural support to tissue constructs.

**TABLE 3:** Relevant studies that have utilized microspheres in 3D bioprinting applications.

References	Encapsulated cell or drug	Microsphere composition	Dispersed phase solvent	Continuous phase	Cross linker	Method	Application	Findings
Levato et al. (2014)	Bone marrow- derived mesenchymal stem cells (BMSCs) from Lewis rats	PLA	EtLac	70% EtOH with 0.3% PVA	n/a	Dispersed through dual concentric nozzle into bath	Bilayered osteochondral models with BMSC-laden PLA microcarriers encapsulated into gelMA-gellan gum bioinks for bone compartment	Microspheres improved compressive modulus of the constructs, facilitated cell adhesion, supported osteogenic differentiation and bone matrix deposition by BMSCs
Poldervaart et al. (2014)	Vascular endothelial growth factor (VEGF)	10 w/v% gelatin	Water	Refined olive oil	10.6 mM glutaraldehyde	W/O emulsion	Controlled release of VEGF from gelatin microparticles to prolong VEGF activity and promote vascularization of endothelial progenitor cells within 3D bioprinted scaffolds	Release of VEGF was continuous for 3 weeks, microspheres enabled higher vessel formation
(Tan et al., 2016)	L929 fibroblasts	PLGA with ethanolic NaOH treatment to increase pore size	DCM	PBS then 0.3 w/v% PVA	n/a	W/O/W double emulsion	Cell-laden microspheres encapsulated in a collagen-agarose hydrogel for bioprinting	Microspheres reduced required initial cell density required for bioprinting, improved mechanical strength of bioink
Xin et al. (2019)	n/a	PEG	DTT	Light mineral oil with Span 80 (0.5 wt%)	LAP	W/O electrospray	Human mesenchymal stem cells mixed with inoculated on PEG microspheres (termed microgels) of varying stiffness with RGD peptide and used as bioink	Cells can spread and proliferate in interstitial spaces between microspheres., Highly tunable and printable into complex structures
Xu et al. (2019)	3T3 fibroblasts	0.5–2 w/v% sodium alginate	DMEM	2 w/v% CaCl_2_	2 w/v% CaCl_2_	Inkjet-based bioprinting using a piezo actuator	Cell-laden microsphere based bioink	Higher concentrations of sodium alginate increased cell viability
Abu Awwad et al. (2020)	GET-RUNX2 protein	PLGA	DCM	PBS	0.25 w/v% methylcellulose	S/O/W emulsion	Sustained release of exogenously delivered transcription factor RUNX2 in mechanically-strong 3D printed biomaterial for bone tissue implants engineering	Controlled release of GET-RUNX2 from microspheres improved osteogenic differentiation
Sharma et al. (2020)	Guggulsterone	PCL	DCM	2% PVA	n/a	O/W emulsion	Drug-laden microspheres incorporated into a bioink for neural induction of hiPSC-derived NPCs to create neural tissue models	Microspheres preserved cell viability and promoted neural differentiation of NPCs
De La Vega et al. (2021)	Puro and retinoic acid	PCL	DCM	2% PVA	n/a	O/W emulsion	Drug-laden microspheres incorporated into a bioink for neural induction of hiPSC-derived NPCs to create neural tissue models	Microspheres improved NPC differentiation into mature neuronal subtypes
Li et al. (2021)	Kartogenin	PLGA	DMSO and DCM	1% PVA	n/a	O/W emulsion	Sustained release of KGN in 3D printed PCL/decellularized meniscus ECM scaffold seeded with synovium-derived MSCs for generation of meniscus tissue	Microspheres improved chondrogenic differentiation of synovium-derived MSCs
Sharma et al. (2021b)	Guggulsterone	PCL	DCM	2% PVA	n/a	O/W emulsion	Drug-laden microspheres incorporated into a bioink for neural induction of hiPSCs to create neural tissue models human induced pluripotent stem cells	Microspheres improved mechanical strength and printability of bioprinted constructs
Bai et al. (2022)	BMP-2, TGF-β, or bFGF	PLGA	Chloroform	1% PVA for primary W/O, 0.5% PVA for double emulsion	n/a	W/O/W double emulsion via ultrasonication	Sustained release of tenogenic, chondrogenic, and osteogenic growth factors via microsphere carriers embedded in layered bioprinted constructs for enthesis tissue engineering	Region-specific differentiation of stem cells *in vitro*, enhanced the enthesis regeneration in a rabbit rotator cuff tear model
Bonany et al. (2022)	n/a	15 wt% gelatine	ddH_2_O	Olive oil	50 mM EDC and 75 mM NHS	W/O emulsion	Microspheres incorporated into alginate-based bioinks to improve cell adhesion and to improve their mechanical properties of the bioink	Microspheres improved rheological properties of bioink, enhanced cell proliferation and osteogenic differentiation
Cohen et al. (2022)	Co- encapsulation of alveolar macrophages and epithelial cells	Polyethylene glycol- fibrinogen	1 v/v% of 10 w/v% pluronic F68, 1.5 v/v% triethanolamine, 0.39 v/v% of N-vinyl pyrrolidone	Mineral oil	1% (v/v) of 10 mM eosin Y photo-initiator	W/O emulsion	Delivered in airway-on-chip model developed from 3D printed molds	Protected cells from LPS exposure (preserved high viability and secreted only moderate levels of TNFα)
Xie et al. (2022)	Human umbilical vein endothelial cells (HUVECs)	15% (w/v) gelatin	PBS	Silicon oil	Thermo- cross- linked	Electrospray	Cell-laden sacrificial microspheres encapsulated in a GelMA-based bioink (applied to create vascularized breast tumor tissue with breast tumor cells embedded in bioink)	Microspheres increased cell viability and proliferation of 3D printed cells through creation of porous network, and enabled vascularization of scaffold
Wang et al. (2022b)	n/a	3% CSPO or 3% PG	Deuterated water	Petroleum ether containing span 80	n(AA): n(NHS): n(EDC • HCl)	O/W emulsion	Microspheres incorporated into biomaterial for increased strength for bioprinting	Composite hydrogel with microspheres had superior storage modulus, shear-thinning and self-healing ability, good extrudability and fidelity
Wen et al. (2022)	Granulocyte colony- stimulating factor (G-CSF)	Protein-dextran-PLGA	DCM	1% PVA and 5% NaCl	n/a	Solvent evaporation	G-CSF-loaded microspheres incorporated into gelatin/alginate bioink used to promote endometrial regeneration in a SD rat intrauterine adhesion model	Microspheres promoted local endometrial regeneration
Chu et al. (2023)	RSC96 Schwann cells	Decellularized extracellular matrix	DMEM	fluorinated carbon oil stabilized by a biocompatible triblock perfluorinated copolymer surfactant (0.5 w/v%, PEG-Krytox- PEG)	0.5% w/v, PEG-Krytox-PEG	W/O droplet based microfluidic chip	Composite bioink consisting of HUVEC-laden GelMA as continuous phase and decellularized extracellular matrix cell-laden microgels as the discrete phase	Microspheres provided extracellular matrix-like microenvironment for cell encapsulation, and considerable shear-resistance to improve post-printing cell viability
Mirek et al. (2023)	Rhodamine or ampicillin	15% PCL or 15% PES	PCL in DMF and PES in NMP	Polymer non- solvent	n/a	Electrospray with wet phase inversion	Microspheres incorporated into GelMA/gelatin biomaterial for antibacterial activity	Addition of microspheres improved structure, thermal, and drug delivery properties
Ou et al. (2023)	*Escherichia coli* DH5α, A549, and HEK 293 T cells	Shell: 5% gelatin and 10% gelMA Core: cell-laden 1% CMC	PBS	Novec 7,500 fluorocarbon (3 M) containing 0.1 v/v% pico-surf surfactants	Shell: 0.5% LAP Core: 10 U/ml TG	Flow- focusing microfluidic chip	Core-shell microgel ink for bioprinting	Mitigated cell leakage and created a favorable environment for cell culture
Reynolds et al. (2023)	n/a	2.0 w/v% gelatin, 0.25 w/v% Pluronic F-127, and 0.1 w/v% chitosan	51.5 v/v% ethanol	n/a	n/a	Coacervation method	Integration of microporogen-structured matrix tailored by adding sacrificial gelatin- chitosan microparticles into a 3D tumor model	Printed tumor cells remained viable and proliferated, while antigen-specific cytotoxic T cells were able to migrate to the tumor site in the matrix to and induce cell death
De Barros et al. (2023)	Insulin-like growth factor-1 (IGF-1)	PLGA	DCM	1% PVA	n/a	O/W in microfluidic chip	Sustainable release of IGF-1 for *in vitro* muscle engineering	Successful adsorption of IGF-1 and sustained release of IGF-1 at physiological pH, promoted the alignment of myoblasts and differentiation into myotubes
Yin et al. (2023)	Chondrocytes from articular cartilage of SD rats	GelMA and alginate	Deionized water	Liquid paraffin with span 80	CaCl_2_	Microfluidic chip	Composite bioink containing microsphere- embedded chondrocytes for 3D printing multiscale scaffolds for articular cartilage repair	Biocompatible *in vivo*, supported cell proliferation and differentiation
Mclean et al. (2024)	n/a	Thiol-ene- based	1,2-dichloroethane with surfactant Hypermer B246	Ultrapure water	Photoinitiator diphenyl (2,4,6-trimethylbenzoyl) phosphine oxide/2-hydroxy-2-methyl-propiophenone blend	W/O emulsion	Composite printable biomaterials composed of nanofibrillar cellulose or gelMA mixed with varying amounts of porous polyHIPE microparticles to tune mechanical properties	Varied ratios of polyHIPEs enable composite hydrogels that mimic mechanical properties of neural tissue (0.1–0.5 kPa), liver (1 kPa), lungs (5 kPa), and skin (10 kPa) while maintaining biocompatibility
Zheng et al. (2024)	BMSCs from rats overexpressing Usp26	5 wt% GelMA	Water	Paraffin oil with 5 v/v% span 80	0.5 wt% LAP	Microfluidic chip	GelMA microspheres loaded with BMSCs and seeded into PCL 3D printed scaffolds to repair bone defects	Model accelerates intervertebral bone fusion, microspheres promote adhesion, and ensures cell activity and Usp26 supplementation

AA, adipic acid; BMSCs, bone marrow derived mesenchymal stem cells; CaCl_2_, calcium chloride; CMC, carboxymethylcellulose; CSPO, hydroxy propyl chitosan; DCM, dichloromethane; DMEM, Dulbecco’s modified Eagle medium; DMF, dimethylformamide; DMSO, dimethyl sulfoxide; DTT, dithiothreitol; ECM, extracellular matrix; EDC, 1-ethyl-3-(3-dimethylaminopropyl) carbodiimide; GelMA, gelatin methacrylate; hiPSC, human induced pluripotent stem cell; HCl, hydrogen chloride; IGF, insulin-like growth factor; KGN, kartogenin; LAP, lithium phenyl-2,4,6-trimethylbenzoylphosphinate; MSCs, mesenchymal stem cells; NaCl, sodium chloride; NaOH, sodium hydroxide; NHS, n-hydroxysuccinimide; NMP, n-methyl-2-pyrrolidone; NPC, neural progenitor cell; O/W, oil-in-water; PBS, phosphate buffer solution; PCL, polycaprolactone; PEG, polyethylene glycol; PES, polyethersulfone; PG, polypropylene glycol; PLA, polylactic acid; PLGA, poly lactic-co-glycolic acid; polyHIPEs, polymeric high internal phase emulsions; PVA, poly(vinyl alcohol); RGD, arginylglycylaspartic acid; S/O/W, solid-in-oil-in-water; SD, sprague-dawley; TG, transglutaminase; VEGF, vascular endothelial growth factor; W/O, water-in-oil; W/O/W, water-in-oil-in-water.

### 4.1 Microspheres for drug delivery

The use drug releasing microspheres for bioprinting application is rapidly becoming one of their applications. Drug releasing microspheres enables delayed or sustained release of the drug in the target area by diffusion, degradation, or in response to environmental factors such as pH change. In this context, the term drug is used as a general description of an additional material encapsulated or attached to the microsphere; this material can include pharmaceutical drugs, cell media, growth factors, or other materials that can enhance the efficacy of a scaffold used in a medical treatment or an experimental model (Mirek et al., 2023). The drugs can be loaded/encapsulated into the microspheres either during (Li et al., 2021) or after microsphere creation (Chen et al., 2020). Additionally, the drug containing microspheres can be integrated with a 3D printed scaffold before bioprinting by incorporating them into the biomaterial (Wen et al., 2022), or after through injecting the microspheres directly into the scaffold (Li et al., 2021). Most drug delivery systems using microspheres and bioprinting rely on diffusion and degradation mechanisms. The initial release is from the diffusion of the materials from the interior/surface of the microsphere into the surrounding tissue/fluid and the later stage release is from the degradation of the structure of the microsphere (Gu et al., 2016). Degradation mediated cargo release often results from prolonged exposure to typical physiological conditions prolonged exposure to physiological conditions (De Barros et al., 2023; Mirek et al., 2023), but can also be modified to release drugs at a specific pH (Wang N. et al., 2023). By controlling the release of drugs through the use of microspheres can maintain drug concentrations within target ranges and diminish side effects caused by concentration extremes and repeated administrations (Wu et al., 2011).

Drugs contained within microspheres diffuse into the surrounding environment and can allow for structural support of the surrounding tissue in addition to providing increased tissue regeneration from the sustained release of drugs. In a study by Li et al. (2021), kartogenin-loaded poly(lactic-co-glycolic) acid (KGN) microspheres were injected into a 3D porous poly(e-caprolactone)/meniscus extracellular matrix (PCL/MECM) scaffold in order to improve meniscus regeneration after injury. The scaffold, composed of PCL, was printed and then injected with MECM and the KCL microspheres (created using O/W emulsion) before being surgically implanted into the knee joint. The KGN was released from the scaffold in a first wave (60%) over the first three days followed by a slow continuous release until day 28. KGN release had minimal effect on cell viability of the surrounding tissue and increased healing of the meniscus. Wen et al. (2022) incorporated granulate colony-stimulating factor (G-CSF) laden microspheres into the biomaterial prior to bioprinting and created a hydrogel scaffold to treat intrauterine adhesions. The PLGA microspheres were prepared using solvent evaporation prior to adding them to the bioink. Once implanted into the rat uterus, the scaffold supported a sustained release of the drug over 40 days, with the highest burst in the first 2 days. Another example is for the stimulation of vascularization using vascular endothelial growth factor (VEGF) loaded in gelatin microspheres within 3D bioprinted scaffolds containing endothelial progenitor cells (Poldervaart et al., 2014). Scaffolds with VEGF laden gelatin microspheres were shown to improve vessel formation and the number of perfused vessels *in vivo*.

**FIGURE 5:**
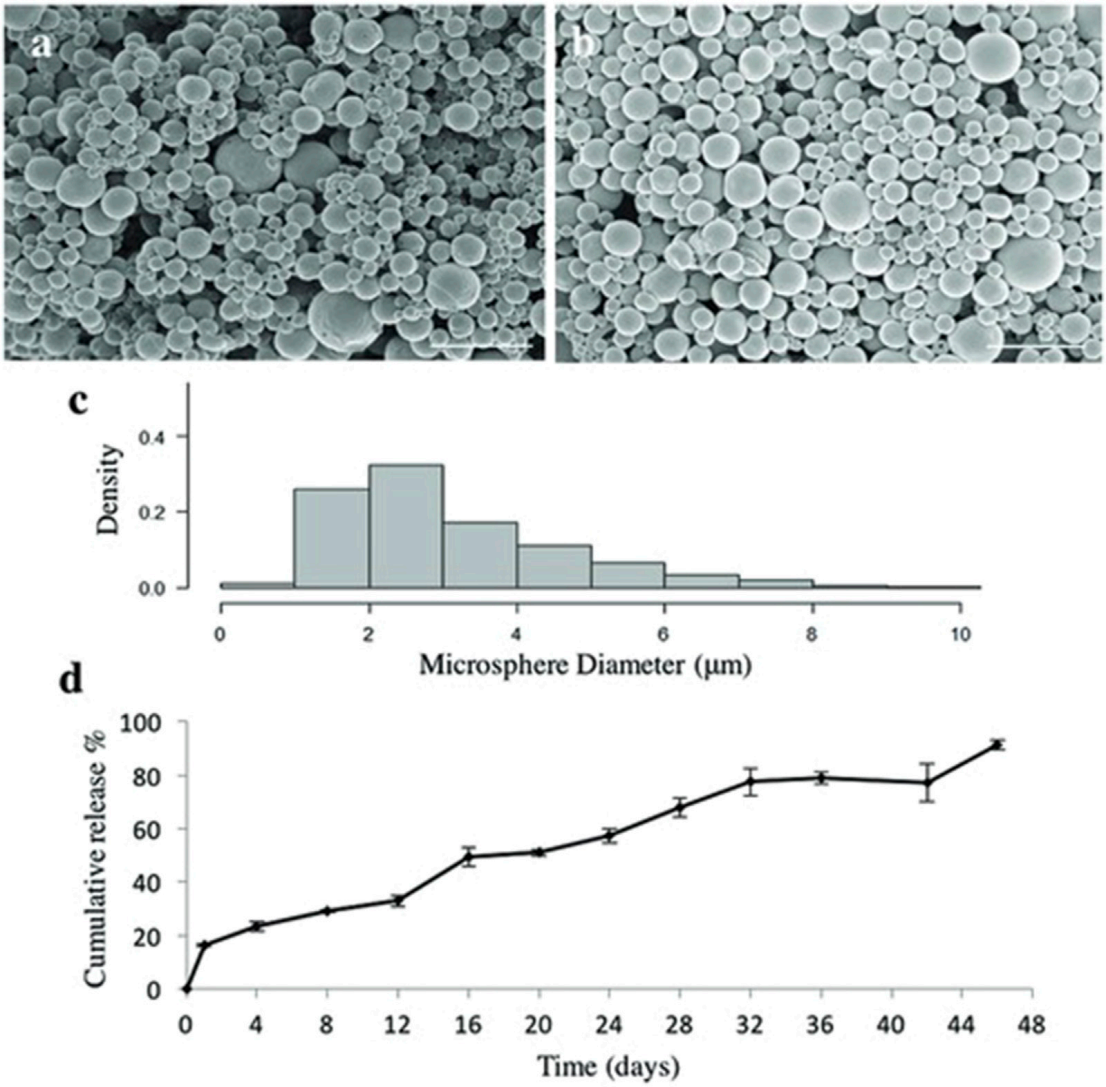
Characterization of purmorphamine loaded microspheres. **(a)** Scanning electron microscopy images showing shape and size of puro-loaded microspheres which have an average size of 3.4 ± 1.17 µm, n = 600, **(b)** and blank microspheres with an average size of 2.24 ± 2.04 µm, n = 600. Scale bars are 10 µm. **(c)** Density histogram showing the distribution of the measured microsphere diameters for purmorphamine-loaded microspheres. **(d)** Quantification of purmorphamine release over 46 days (n = 3)– (91%) of the drug was released at the end of the time period. The data are being reported as the average with the error bars representing the standard deviation. Taken from (De La Vega et al., 2018) and reprinted with permission.

**FIGURE 6:**
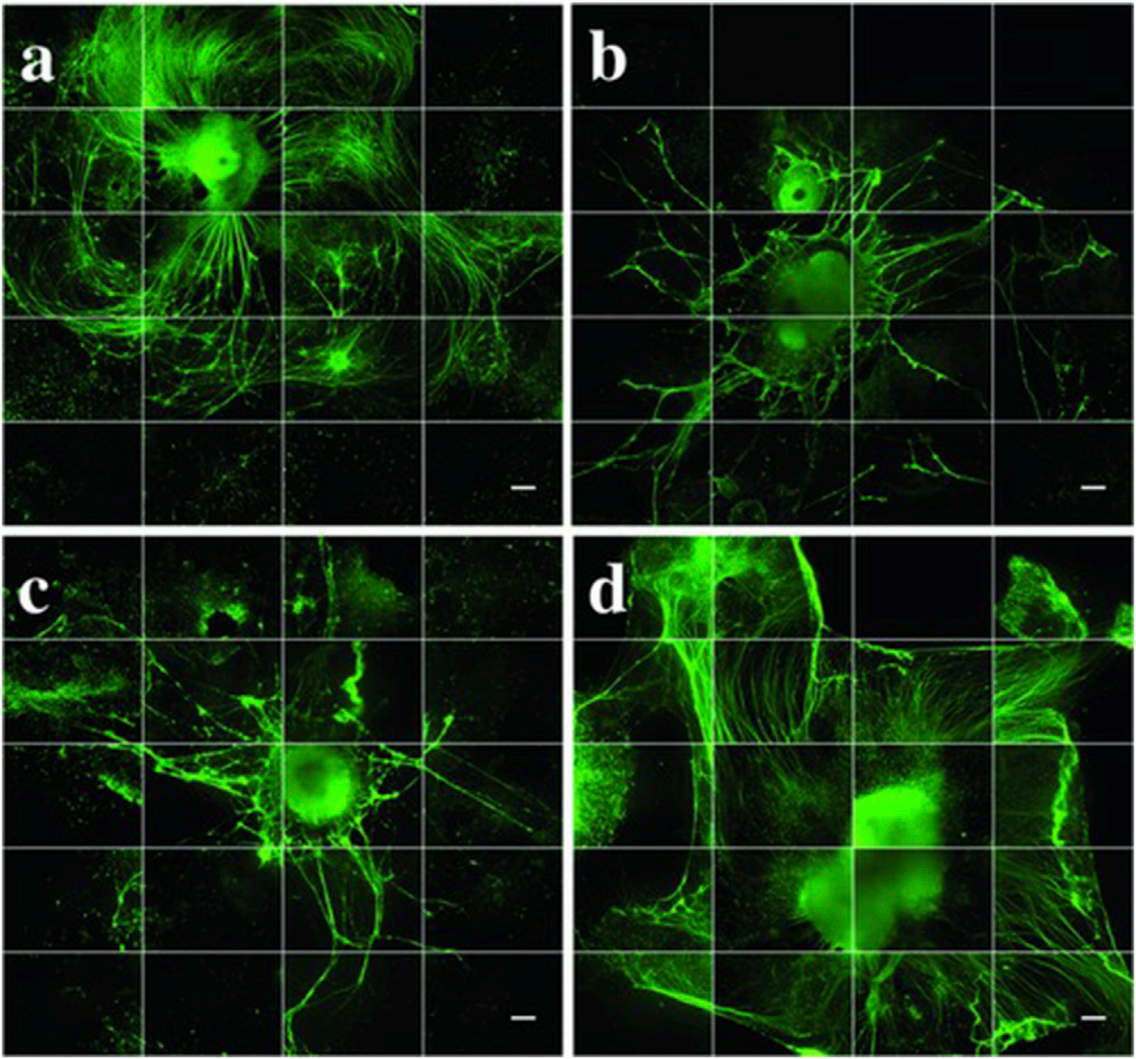
Neurite growth extension after 35 days *in vitro*. All groups (n = 3, with 1–5 aggregates per well) were stained for β-tubulin III (βT-III neuronal marker, green. **(a)** puro/RA-loaded-microspheres (M), **(b)** unloaded microspheres (U), **(C)** negative control (N) and **(d)** positive control (P). Images are made into montages with ImageJ software from 36 images taken by an IncuCyte automated imaging machine. Scale bars represent 300 µm. Taken from (De La Vega et al., 2018) and reprinted with permission.

Sharma et al. (2020) added guggulsterone encapsulated PCL microspheres in their bioink to improve neural differentiation of human induced pluripotent stem cell-derived neural progenitor cells (NPCs). The NPCs were incorporated into a bioink consisting of fibrinogen, alginate, and genipin dissolved in dimethyl sulfoxide (DMSO). It was found that the NPCs expressed early markers of dopaminergic neural differentiation without adverse effects on neurite length and branching when exposed to the guggulsterone-loaded microspheres (Sharma et al., 2020). Further work by De la Vega et al. (2018), De la Vega et al. (2021), encapsulated purmorphamine and retinoic acid in PCL microspheres in a fibrin based bioink to engineer neural tissue and were shown to increase the cell viability and neural differentiation. Figure 5 from De La Vega et al. (2018) shows the size and release dynamics of the purmorphamine-loaded microspheres. Figure 6 shows the effect of the loaded microspheres on neurite growth extension after 35 days *in vitro* (De La Vega et al., 2018). Similarly, Bai et al. (2022) used PLGA microspheres for sustained release of tenogenic, chondrogenic, and osteogenic growth factors in printed living tissue constructs to improve rotator cuff injuries. The PLGA microspheres were prepared using double emulsion before being added to the bioink and printed into a porous scaffold. The scaffold was then implanted onto the end of a tendon stump where it degraded over 12–18 days to reduce the formation of fibrovascular scar tissue and facilitate the reconstruction of the injured tendon.

Drugs encapsulated within microspheres can also be released due to external environmental factors such as pH. A specific pH of the surrounding tissue can trigger the microspheres to degrade, allowing for the encapsulated drug to be released. This method can be seen in De Barros et al. (2023) where insulin-like growth factor (IGF)-1 was encapsulated in PLGA microspheres and mixed in with a biomaterial before 3D bioprinting in order to promote the alignment of myoblasts and myotubes. IGF-1 had a sustained release over 21 days at physiological pH which had a regenerating effect on surrounding muscle tissues. There are few studies published within the field of bioprinting that use pH sensitive microspheres; however, the method is worth further discussion. For example, the use of pH-sensitive PLGA microspheres could be used to release encapsulated drugs at lower pH (i.e., 5.5), which is beneficial for drug release in acidic environments such as the stomach, tumors, and areas of inflammation, enabling targeted delivery to affected areas (Wang N. et al., 2023).

When producing microspheres for drug delivery, important considerations are the drug encapsulation efficiency and the rate of drug release. To enable consistent and predictable drug release, methods such as cross-flow membrane emulsification or microfluidic techniques that produce consistently sized microspheres may be beneficial (Hancocks et al., 2013). Combining more complex methods that generate consistent particles with principles of W/O/W double emulsions enable higher encapsulation efficiency of hydrophilic drugs, and will protect the drug against light, enzymatic degradation, and oxidation due to the additional emulsion step. This additional step enables the organic layer to act as a barrier that prevents the hydrophilic drug from diffusing into the external aqueous phase (Iqbal et al., 2015). However, depending on the scale of production, some methods may be cost prohibitive. Lower complexity mechanical agitation methods may be used to reduce cost, but depending on the desired size of the microsphere, may be limited by high levels of shear stress that can damage the microspheres and the drugs during production. They can further be limited by the use of cytotoxic solvents and co-solvents, though this is dependent on the solubility of the chosen polymer and drug (and may still be a limitation in other production methods). Drug delivery microspheres created using electrospray can result in highly tunable microspheres without the use of harsh solvents and the encapsulation efficiency is dependent on the solubility of the polymer and/or drug being used (Morais et al., 2020).

### 4.2 Cell laden microspheres

Cell therapies, and specifically stem cells, are increasingly of interest for the treatment of a variety of conditions due to their reparative and immunomodulation capacities in wound healing and tissue regeneration, and their high proliferation rates and multipotency that enable them to be expanded and differentiated into specific cell types (Ong et al., 2018). Injecting cells directly is limited by their rapid dispersion and clearance from the target area (Li et al., 2021). For this reason, the use of hydrogels for direct injection has been investigated; however, hydrogels provide a highly hydrophobic microenvironment constraining the suspended cells to a round shape, regardless of their native morphology. Moreover, most injectable hydrogels have limitations such as having sufficient mechanical stability and durability to support cell proliferation and differentiation before the formation of new tissue, in addition to being too soft for applications in load-bearing regions of the body, further limiting their application. (Levato et al., 2014; Liao et al., 2017). Bioprinting can offer a solution to this problem, as it offers more control over structure and cell density; more specifically with the use of stem cells or other cell types to create bioprinted tissues for implantation, as well as tissue models, organoids, and organs-on-a-chip that can be used for treatment testing and/or to better understand disease pathology (Ong et al., 2018). However, there are challenges that arise when trying to print with cells mixed directly into the bioink. Many bioprinters struggle to maintain cell viability due to their method of crosslinking (e.g., lasers, UV exposure, cytotoxic photo initiators) (Bakhshinejad and D’souza, 2015; Liao et al., 2017), in addition to difficulty maintaining the desired cell density throughout the constructs. For this reason, extrusion-based printers are most commonly used; however, cells are often subjected to high levels of shear stress as they are pushed through the nozzle, leading to an increase in cell death at higher pressures. In addition, it does not ensure that cells are distributed evenly throughout the construct (Vanaei et al., 2021).

One solution that shows promising results is to encapsulate the cells inside of microspheres, termed cell laden microspheres (CLMs), which can protect the cells from high shear forces that occur during extrusion (Tan et al., 2016; Xu et al., 2019; Xie et al., 2022; Chu et al., 2023; Yin et al., 2023). CLMs are porous microspheres that encapsulate cells and allow them to adhere and proliferate prior to bioprinting (Tan et al., 2016). The encapsulation of the cells provides a layer of protection from the external forces they are exposed to during bioprinting. The encapsulation process also allows the concentration of cells to be controlled, while the printed construction minimizes the unwanted dispersion of cells through surrounding areas. The material chosen for the microsphere itself varies depending on the desired application and cell type; however, the principal characteristics that are important to consider are consistent and include the degradation rate, viscosity, and impact on cell viability.

Microspheres can first be made, then used to seed cells, as was done by Levato et al. with their mesenchymal stromal cell (MSC)-laden polylactic acid microspheres; or used as microcarriers in bioreactor production systems, which can then be incorporated directly into the bioinks (Levato et al., 2014). The microcarriers give adherent cells a surface to attach to and proliferate on in suspension culture (which most bioreactors require) (Schnitzler et al., 2016). However, CLMs are typically created by encapsulating the cells within the microsphere as it is being formed. In the simplest form, and most commonly, CLMs are homogenous, consisting of a single cell type and a single material. Yin *et al.* found that by using a microfluidic chip to encapsulate chondrocytes in GelMA/alginate microspheres, they were able to create a 3D printed composite scaffold, which when implanted into rats, resulted in the formation of cartilage tissue with uniform distribution throughout the construct (Yin et al., 2023).

Cells can also be encapsulated as core-shell microspheres, with all the cells being concentrated within the inner core of the microsphere and coated with a porous external shell layer. For example, gelMA and carboxymethylcellulose (CMC) core-shell microspheres were generated using a microfluidic device to encapsulate human embryonic kidney (HEK) 293 T cells in a CMC core, and the CLMs were then printed using an extrusion-based 3D bioprinter (Ou et al., 2023). It was found that encapsulating cells in core-shell microspheres improved the cell viability and further prevented cell leakage in comparison to non-core-shell microspheres (Ou et al., 2023).

**FIGURE 7:**
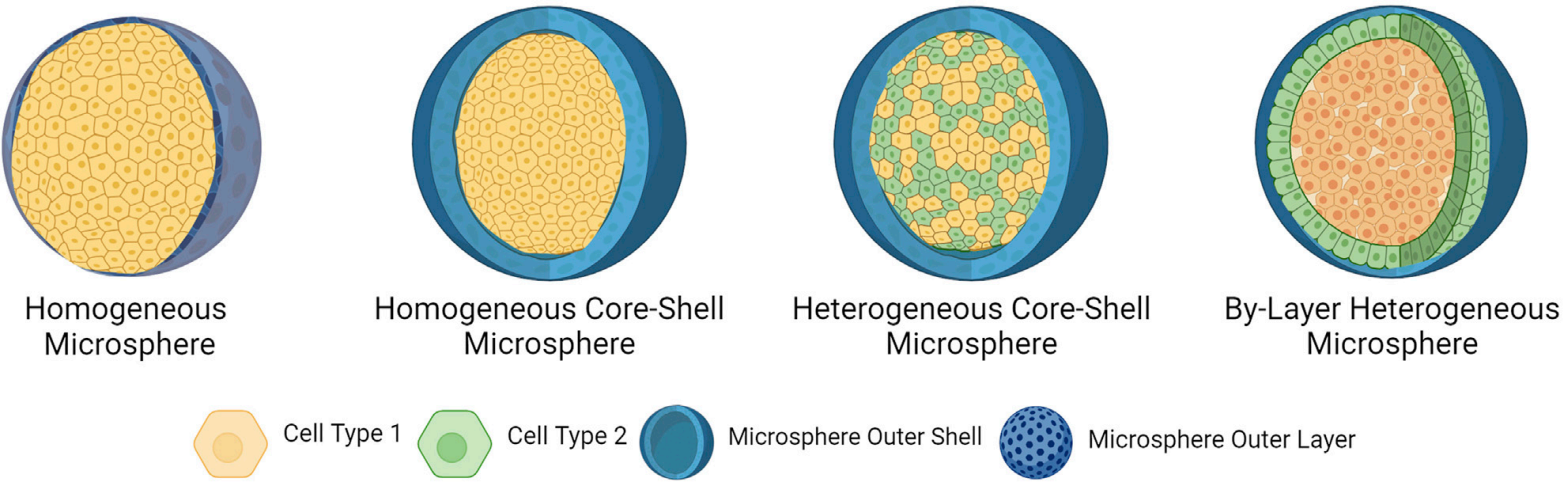
Types of cell-laden microspheres. Homogeneous microspheres are porous hydrogels incorporating a single cell type. Homogeneous core-shell microspheres encapsulate a single cell type within its core and are coated in a porous external shell layer. Heterogeneous core-shell microspheres encapsulate multiple cell types within the core and are coated in a porous external layer. By-layer heterogeneous microspheres incorporate one cell type in the external layer, and a second cell type encapsulated in the core. Created in BioRender. Willerth, S.M. (2024) https://BioRender.com/c94d555.

CLMs can also be used as a means of co-culturing cells. In some applications, such as for organ-on-a-chip or wound repair, it is beneficial to utilize multiple cell types that could induce synergic biological effects. In such scenarios, two or more cell types are co-encapsulated inside a microsphere carrier, resulting in a heterogeneous CLM (Cohen et al., 2022). Co-encapsulation of CLMs is typically done in one of two ways: either by having multiple cell types encapsulated together within the microsphere, or by encapsulating different cell types in bilayer core-shell microspheres. A visual representation of the different types of CLMs is shown below in Figure 7. While there have been limited examples of their use in the context of bioprinting, it has been used to create organ-on-a-chip models and showed improvements in biomimetics, cell viability, and proliferation; offering promise for future bioprinting applications (Cohen et al., 2022; Liu et al., 2022; Wu et al., 2022). Using a microfluidic chip, Chen *et al.* were able to create GelMA/chitosan microspheres co-encapsulating PC12 and RSC96 Schwann cells; which were then 3D bioprinted into a composite scaffold, enabling them to mimic the epineurium layer (Chen et al., 2020). Cohen et al. created heterogeneous CLMs through membrane emulsification using alveolar macrophages and epithelial cells for the therapeutic delivery of immune cells to an airway-on-chip platform (Cohen et al., 2022). Similarly, Wu et al. co-encapsulated HepG2, HUVEC, and HFF-1 cells in sodium-alginate microspheres using electrospray to improve the parallelism of liver-on-chip models for drug screening (Wu et al., 2022). Liu et al. created heterogeneous core-shell microspheres by embedding hepatocytes into the inner layer and hepatic stellate cells in the outer layer to form orderly liver structures for improved biomimetics of liver-on-a-chip platforms used in drug development (Liu et al., 2022).

Considerations for determining methods to create cell laden microspheres are degradation rate and the impact on cell viability and functionality. Cells are responsive to their external environment, and the material must be optimized for stiffness, cell attachment, and preserving or promoting their desired function. For producing microspheres, the forces they are exposed to during the process will highly determine what methods can be used. Mechanical agitation and extrusion through membrane filters create high levels of shear stress, and the solvents and co-solvents must be chosen carefully to avoid interactions with potentially cytotoxic materials. Microfluidics is the most common method to generate cell laden microspheres, where the adjustability of the chip design results in precise, highly tunable, and reproducible microspheres, and channels can be designed to reduce shear. In summary, encapsulating cells in microspheres to create CLMs represents an important area of development in the world of 3D bioprinting that shows promise as a solution to the common challenges of cell viability and cell dispersion when developing bioprinted tissues.

### 4.3 Microspheres for structural applications

Tailoring mechanical properties of bioinks to match the properties of native tissues remains challenging (Yu et al., 2018; Parak et al., 2019). The stiffness or mechanical strength of 3D printed scaffolds is of particular importance due to the impact on cell growth, proliferation, and function grow and proliferate (Ouyang et al., 2016; Paxton et al., 2017; Chopin-Doroteo et al., 2021). Different tissue types possess different structural strength requirements. For example, bone cells (osteocytes) require a much stiffer extracellular environment than neural tissue (i.e., 100 kPa compared to 1 kPa) (Heath and Cooper, 2017; Samanipour et al., 2022; López-León et al., 2023). However, it is often difficult to optimize the structural strength of a particular bioink while also maintaining biocompatibility (non-toxicity and non-immunogenicity) and biodegradability (Ouyang et al., 2016; Sharma et al., 2021a). One solution is the use of microspheres as structural supports in bioinks, as they may still be created from biocompatible and biodegradable materials but provide a stiffer overall structure when compared to the conventional method of crosslinking hydrogels. Because microspheres are discrete units and not a solid barrier, their incorporation into a hydrogel scaffold does not compromise the diffusion rates of nutrients and oxygen throughout the construct, which is necessary for cell growth. Microspheres also provide a solid structure for cells to attach to, and this cell adhesion in turn promotes proliferation (Bonany et al., 2022).

Early studies revealed that biomaterials containing microspheres possessed improved mechanical properties when printed. Levato et al. (2014) added cell-laden polylactic acid (PLA) microspheres to a gelatin methacrylamide-gellan gum biomaterial ink. While printing a bilayered bone model, they observed an increased compressive modulus and improved printability, while at the same time improving cell adhesion and osteogenic differentiation. In a similar study, Tan et al. (2016) printed cell-laden PLGA microspheres in an agarose-collagen bioink. They found that the microsphere-containing bioink had over 100 times the mechanical strength, when compared to the agarose-collagen bioink on its own. In addition, the PLGA microspheres were created to be highly porous, which was found to increase cell adhesion and proliferation.

Past research has also examined the mechanical properties of bioinks containing drug-encapsulated microspheres. For example, Sharma et al. (2021b) sought to mimic the mechanical properties of neural tissue. As previously mentioned, they used a fibrin-based bioink and PCL microspheres containing the drug guggulsterone to stimulate the differentiation of NPCs into dopaminergic neurons. In addition to the advantages of drug encapsulation, they found that the microsphere-containing bioink possessed significantly higher mechanical strength and lower degradability, which was conducive to a healthy environment for neural cells. The printed construct also had a higher elastic modulus that was approximately equivalent to the adult human brain (800–1,400 Pa) (Budday et al., 2020; Kren et al., 2024), when compared to a construct of identical composition without the addition of microspheres (1,032 ± 59.7 Pa vs. 728 ± 47.6 Pa).

**FIGURE 8:**
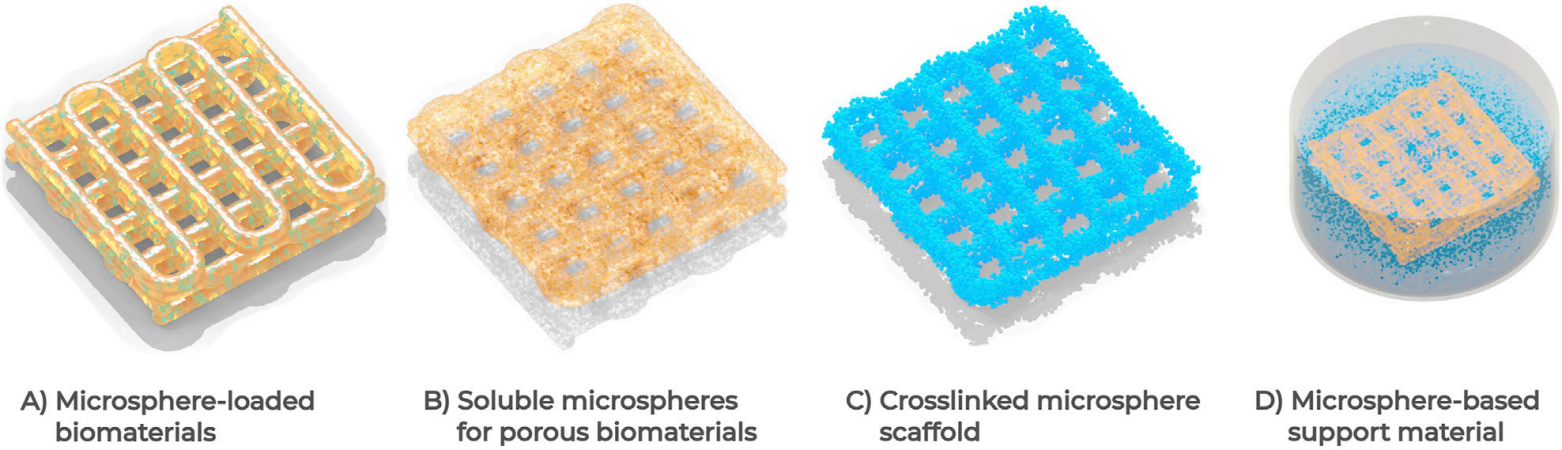
Visual comparison of structural microsphere applications. **(A)** Loading the biomaterial with microspheres before or after printing, resulting in a microsphere-infused construct. **(B)** Loading a biomaterial with soluble microspheres, creating a porous and vascularized construct. **(C)** Crosslinking microspheres directly to create a printable biomaterial, the structure is formed with microspheres rather than a base material. **(D)** Microspheres are used as a support material (e.g., FRESH V2.0) and are not part of the construct.

Researchers have also examined the use of microspheres as structural components of biomaterial inks, without the encapsulation of cells or drugs. For example, Bonany et al. (2022) added gelatine and hydroxyapatite microspheres to alginate biomaterial ink containing osteoblasts and observed improved rheological properties, while also providing a protective environment for the cells during extrusion. The increased stiffness enhanced cell proliferation and osteogenic differentiation and could be further tailored by altering the concentration of calcium crosslinker added to the bioink. Mclean et al. (2024) added porous polymeric high internal phase emulsion (polyHIPE) microspheres to a nanofibrillar cellulose or gelMA bioink to tune mechanical properties. They were able to mimic mechanical properties of neural tissue (0.1–0.5 kPa), liver (1 kPa), lungs (5 kPa), and skin (10 kPa) by varying the concentration of polyHIPE microspheres. The common procedure of loading microspheres into biomaterials is shown in Figure 8A.

Sacrificial microspheres have also been added to constructs as a means of creating porous structures and enabling the formation of vascularized networks. For example, gelatin-chitosan microspheres were added into a biomaterial for a 3D tumor model, which degraded upon exposure to physiological conditions and created a microporogen-structured matrix (Reynolds et al., 2023). This method is demonstrated in Figure 8B. In addition, Wang P. et al. (2022) recently developed a technique to chemically crosslink gelatin microspheres, which they used to create a vascularized bone tissue model. The loose-packed structure of the scaffold, visualized in Figure 8C, was beneficial in enabling cell migration and, upon surgical implantation into a mouse femur, the scaffold successfully demonstrated angiogenesis. The idea of sacrificial microspheres has also been used to incorporate cells into printed tissues. Xie et al. (2022) embedded cell-laden gelatin microspheres into a gelMA-based biomaterial ink, where the microspheres were able to increase viability upon printing, and enable proliferation and migration of the cells after the microspheres quickly degraded to create a porous and vascularized scaffold Figure 8B. Additionally, microspheres have further been utilized as a sacrificial support material coined Fresh V2.0, which saw notable improvements over previous non-microsphere iterations (Lee et al., 2019). This method of creating a microsphere-based support material is seen in Figure 8D.

Creating structural microspheres has the fewest limitations regarding production methods, as they do not contain sensitive materials like cells or drugs. Because of this flexibility, any method can be used in their creation. However, biocompatibility must still be considered due to their eventual incorporation into biomaterials. Mechanical agitation requires minimal equipment to operate, making it accessible to most laboratories without the purchase of specialized equipment, and has been the most used method for creating microspheres in this application to date. The simplest set-up can create microspheres with a magnetic stirring hot-plate and beaker (Zhang and Gao, 2007). Electrospray is another potential method for creating structural microspheres, requiring fewer tunable parameters than microfluidic techniques, and offering a higher degree of control than mechanical emulsion-based methods (Qayyum et al., 2017). If more consistently sized microspheres are desired, at the expense of lower yield, the use of microfluidics may be more desirable (Li et al., 2018). The consistent sizing of microspheres created using membrane emulsification is of great use for structural applications if being used for large scale production, but can be prohibitively expensive for small scale production, especially when using cross-flow membrane emulsification, as it requires more specialized equipment than a premix emulsification system (Hancocks et al., 2013). In summary, while still a relatively new application of microsphere technology, microspheres have been shown to successfully enhance the desired mechanical and structural properties of biomaterials. Further research is needed in this area to understand the specific types of microspheres that may be useful in different tissue models.

## 5 Concluding remarks and future perspectives

Microspheres are a valuable area of research for the creation of advanced biomaterials. They have been utilized for the targeted slow release of numerous drugs, due to their tunable degradation kinetics. Microspheres have also been used to encapsulate cells to improve their viability by protecting them from shear stress and other forces during printing, and limit unwanted dispersion of cells outside of the constructs; in addition to providing a means of co-culturing cells in applications such as wound repair and organ-on-a-chip models. Finally, they have been used for improving rheological properties of materials, as well as for creating artificial vasculature. Mechanical agitation methods were most commonly used when creating both drug delivering and structural microspheres, however, microfluidics were used more often when creating cell-laden microspheres.

Microsphere production methods each have a wide range of variables to consider, and optimization for each application is essential. Mechanical agitation can be used for large scale production and requires minimal equipment but provides less control over final particle size, yielding a more heterogenous population of microspheres. Membrane emulsification improves particle consistency while retaining high throughput, but requires a more complex set up, and changing particle size typically requires new filters. In addition, membranes can be prone to clogging depending on the viscosity of the dispersed phase. Microfluidic chips provide much more uniformly sized microspheres at the expense of lower throughput. They provide the most control compared to other generation methods, allowing fine control of flow rates and fluid interactions through channel design, but their added complexity makes the chips difficult to produce and properly tune, increasing cost of production. Electrospray offers an alternative which provides a narrow size distribution without (in some cases) the need for a surfactant or organic solvent but provides lower encapsulation efficiency This method exhibits low forces on the microspheres, although may be unable to produce microspheres from viscous solutions such as high concentration hydrogels. ATPS provides another method removing the need for organic solvents, although to date it has not been extensively utilized for the application of microspheres in bioprinting. In addition, ATPS literature has often focused on larger-sized microspheres (>100 μm) which may present challenges during the printing process; further research is required to overcome this hurdle.

As research continues to develop, new and improved techniques are being proposed; one such platform is microporous annealed particles (MAPS). This method is currently being explored in the field of wound healing and tissue regeneration, with many studies showing promising results when injecting MAPS containing cells *in vivo*. Further research should be conducted to determine the impact on cell viability, proliferation, and particle dispersion when used in 3D bioprinting. While microsphere production methods have been improved upon in recent years, resulting in increasing yields and more consistent results in terms of size and shape (Hossain et al., 2015), many challenges remain. These challenges are evident in large-scale production of microspheres but also impact small-scale processes. Many of the manufacturing processes involve multiple steps, some of which can take place over days, making it both difficult and expensive to scale up production. To date, few studies have directly compared the results between different production methods. Comparative studies often focus on optimizing a single method of production, such as the use of different membrane materials for membrane emulsification. Direct comparisons between different methods of microsphere production for drug delivery, cell encapsulation and structural applications, while using the same base materials, cells, and drugs to ensure proper reproducibility would be a useful avenue of future research. This would enable researchers to tailor their production approach to their application and desired outcome.

Areas for future research with respect to drug delivery and cell laden microspheres would be to investigate the printing of constructs containing both cell laden and drug delivering microspheres as a means for further improving cell proliferation, differentiation, and viability. Additionally, further research should be done to characterize the properties and effects of using core-shell vs. non-core-shell microspheres for both homogeneous and heterogeneous CLMs. Another avenue for more research is the use of pH sensitive drug delivery microspheres as a means for targeted drug release for conditions that cause a shift in the body’s physiological pH, such as cancerous tumors. In terms of structural microspheres, their potential application in wound healing and for load-bearing tissues such as bone repair should be further explored, as the enhanced rheological properties allow for constructs to closely mimic the native tissue. Another area for more research to be conducted is in the longevity and functionality of microsphere-based scaffolds, specifically in medical implants, but relevant to the broader field of bioprinting. In addition, the issue of microsphere migration in implanted constructs remains a challenge that must be addressed (Liao et al., 2017; Neto et al., 2019).

In summary, the addition of microspheres into 3D printed biomaterials represents a new and growing area of research, with important medical applications. Recent successes in microsphere applications have been discussed in this review, with a focus on applications in drug delivery, cell encapsulation and altering the biomaterial ink structural properties. Challenges remain in the production methods of microspheres, which must be optimized for large scale production, and tailored to specific applications.
