# Correction: Microspheres for 3D bioprinting: a review of fabrication methods and applications

**DOI:** 10.3389/fbioe.2025.1642571

**Published:** 2025-07-18

**Authors:** Dmitri Karaman, Kira Williams, Jolene Phelps, Fynn La Boucan, Gretchen Lewandowski, Kerrin O'Grady, Bosco Yu, Stephanie M. Willerth

**Affiliations:** ^1^ Department of Mechanical Engineering, University of Victoria, Victoria, BC, Canada; ^2^ Division of Medical Sciences, University of Victoria, Victoria, BC, Canada; ^3^ Axolotl Biosciences, Victoria, BC, Canada; ^4^ Biomedical Engineering Program, Department of Mechanical Engineering, University of Victoria, Victoria, BC, Canada; ^5^ Biology Program, Department of Science, University of Victoria, Victoria, BC, Canada; ^6^ Department of Biomedical and Chemical Engineering, Syracuse University, Syracuse, NY, United States; ^7^ BioInspired Syracuse, Institute for Material and Living Systems, Syracuse University, Syracuse, NY, United States; ^8^ School of Biomedical Engineering, Faculty of Applied Science and Faculty of Medicine, University of British Columbia, Vancouver, BC, Canada

**Keywords:** microspheres, bioprinting, biomaterials, emulsification, microfluidics, electrospray, drug encapsulation

Author Jolene Phelps was erroneously assigned to affiliation 3 (Axolotl Biosciences, Victoria, BC, Canada) instead of affiliation 1 (Department of Mechanical Engineering, University of Victoria, Victoria, BC, Canada). Authors Fynn La Boucan, Kerrin O’Grady, and Bosco Yu were erroneously assigned to affiliation 2 (Division of Medical Sciences, University of Victoria, Victoria, BC, Canada) instead of affiliation 1. Author Gretchen Lewandowski was erroneously assigned to affiliation 1 instead of affiliation 4 (Biomedical Engineering Program, Department of Mechanical Engineering, University of Victoria, Victoria, BC, Canada). The correct affiliations have now been added for these authors.

The original version of this article has been updated.

